# Direct Growth of Graphene on Silicon by Metal-Free Chemical Vapor Deposition

**DOI:** 10.1007/s40820-017-0173-1

**Published:** 2017-12-08

**Authors:** Lixuan Tai, Daming Zhu, Xing Liu, Tieying Yang, Lei Wang, Rui Wang, Sheng Jiang, Zhenhua Chen, Zhongmin Xu, Xiaolong Li

**Affiliations:** 1 Shanghai Synchrotron Radiation Facility, Shanghai Institute of Applied Physics, Chinese Academy of Sciences, Shanghai, 201204 People’s Republic of China; 2 University of Chinese Academy of Sciences, Beijing, 100049 People’s Republic of China; 3 Department of Electronic Engineering, Tsinghua University, Beijing, 100084 People’s Republic of China

**Keywords:** Graphene, Silicon, Metal-free CVD, Domain growth

## Abstract

The metal-free synthesis of graphene on single-crystal silicon substrates, the most common commercial semiconductor, is of paramount significance for many technological applications. In this work, we report the growth of graphene directly on an upside-down placed, single-crystal silicon substrate using metal-free, ambient-pressure chemical vapor deposition. By controlling the growth temperature, in-plane propagation, edge-propagation, and core-propagation, the process of graphene growth on silicon can be identified. This process produces atomically flat monolayer or bilayer graphene domains, concave bilayer graphene domains, and bulging few-layer graphene domains. This work would be a significant step toward the synthesis of large-area and layer-controlled, high-quality graphene on single-crystal silicon substrates.

**Electronic supplementary material:**

The online version of this article (10.1007/s40820-017-0173-1) contains supplementary material, which is available to authorized users.

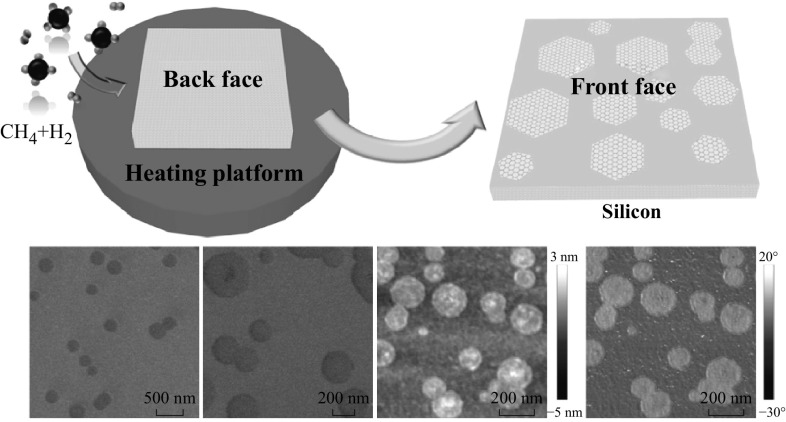

## Highlights

- Graphene was successfully grown on single-crystal silicon substrates using metal-free, ambient-pressure chemical vapor deposition.
- Atomically flat monolayer or bilayer graphene domains, concave bilayer graphene domains, and bulging few-layer graphene domains can be produced by controlling the growth temperature.
- In-plane propagation, edge-propagation, and core-propagation processes are proposed to evaluate the sequentially changing graphene domains.

## Introduction

Graphene is a novel two-dimensional material with remarkable mechanical and electrical properties, such as high carrier-mobility and outstanding mechanical flexibility [1]. Since the first graphene samples were produced via mechanical exfoliation of bulk graphite [2], various synthetic methodologies have been developed for graphene preparation [3–6]. Chemical vapor deposition (CVD) is one of the most efficient methods for the synthesis of large-area graphene films with high crystallinity and large grain size on metal substrates (e.g., copper, nickel) [4, 7]. However, in this conventional method, graphene grown on a metal surface must be transferred to the desired substrate (e.g., dielectrics, semiconductor) for applications. While wafer-scale transfer is possible, this requires complicated and costly transfer procedures, which tend to induce mechanical damage as well as chemical contamination [8]. In other words, it is highly desirable to produce high-quality graphene films on a wide range of substrates directly through CVD without a metallic catalyst.

For the past few years, interest in the direct growth of graphene on semiconductor substrates is increasing [9, 10] due to the unique properties of graphene–semiconductor interfaces, which result in synergetic effects in hybrid devices [11–13]. In particular, silicon plays an important role in the electronic and photoelectronic industries, and the development of hybrid graphene–Si structures/devices may offer a seamless integration of graphene into current microelectronics technology. In fact, graphene–silicon heterostructures have found their way into various applications including Schottky diodes [14], tunable ultrasensitive photodetection [15], and solar cells [16], which would present many more interface properties with the direct growth of graphene on silicon. So far, much research has been dedicated to the direct synthesis of graphene on SiC [17], SiO_2_ [18–22], quartz [23, 24], Al_2_O_3_ [25, 26], Si_3_N_4_ [27], SrTiO_3_ [28], Ge [9, 10], and hexagonal boron nitride (h-BN) [29–31] substrates. Compared to the above substrates, single-crystal silicon has received relatively little attention for direct graphene growth via CVD. This is mainly due to its catalytically inert (compared to metal substrates) nature and the surface instability of single-crystal silicon (compared to SiO_2_, quartz, Al_2_O_3_, Si_3_N_4_, h-BN, etc.) during interaction with active hydrocarbon radicals at high temperature [4, 7, 18–31]. As a result, despite some studies of direct growth on single-crystal silicon substrates using metal-free CVD or solid-source molecular-beam epitaxy, only thick graphite carbon (SiC may be accompanied in these processes) has so far been realized [32–34]. Overall, the quality of graphene directly grown on single-crystal silicon is still far from satisfactory.

In this paper, we demonstrate a novel method for the direct growth of graphene on an upside-down placed, single-crystal silicon substrate using metal-free, ambient-pressure chemical vapor deposition (APCVD). Single graphene domains can be directly nucleated and grown on smooth, single-crystal silicon, regardless of crystal faces and doping levels. To the best of our knowledge, this work presents the first dedicated investigation of graphene growth on single-crystal silicon using metal-free CVD.

## Experimental

### Metal-Free APCVD Growth of Graphene on Silicon Substrates

Single-crystal silicon substrates (500 µm thick, (100), (111), and (110) face, *N*-type, *P*-type, intrinsic silicon, *R*_a_ < 0.5 nm) were purchased from Suzhou Crystal Silicon Electronic & Technology Co. Ltd. A custom-made portable chamber, with a ceramic wafer as a heating platform, was used for the graphene growth. The temperature control was precisely calibrated with an error below ± 0.1 °C. Before placement (upside-down) onto the heating platform, the single-crystal silicon substrates were pretreated by dipping into 15% HF for 15 min, and then cleaned by deionized water and dried by *N*_2_ flow. The CVD chamber was evacuated and back-filled with argon (Ar) three times to create an inert environment. The single-crystal silicon surface was then heated to the desired temperatures (900–930 °C) and stabilized for 10 min under 100 sccm H_2_ and 200 sccm Ar to remove any organic residues and activate the growth sites. Typical graphene growth was performed with a gas mixture of 180 sccm CH_4_ and 10 sccm H_2_ for 1 h. After that, the chamber was cooled down to room temperature in 1 h with 180 sccm of CH_4_ and 10 sccm H_2_. As a comparison, the conventionally placed silicon substrates (faceup) were also used for graphene growth in the same processing conditions as that of the upside-down samples.

### Sample Characterization

Atomic force microscopy (AFM) images were acquired with a Bruker Multimode 8 SPM with NanoScope version 8.15 software and a NanoScope V Controller using a tapping mode. The silicon tip with a nitride cantilever (SNL, 0.35 N m^−1^, Bruker) was treated with a plasma cleaner (Harrick Plasma, Plasma Cleaner PDC-32G) for 2 min prior to use. Raman spectra were recorded at room temperature using a Horiba Scientific LabRAM HR Evolution Raman spectrometer with a 473-nm wavelength laser. The laser spot size was about 2 µm, and the exposure time was 30 s. High-resolution scanning electron microscopy (SEM) images were obtained by Model JSM-7600F (JEOL Ltd.). A monochromatic, Al Kα (*hυ* = 1436.6 eV) X-ray source with a power of 36 W and a spot size of 40 μm was used to collect spectra at a background pressure of ~ 2×10^−9^ torr. X-ray photoelectron spectroscopy (XPS, K-Alpha, Thermo Fisher Scientific Inc.) was used to characterize the samples. For the C 1s XPS spectrum, the binding energies were referenced to the C 1s line at 284.8 eV.

## Results and Discussion

**Fig. 1:**
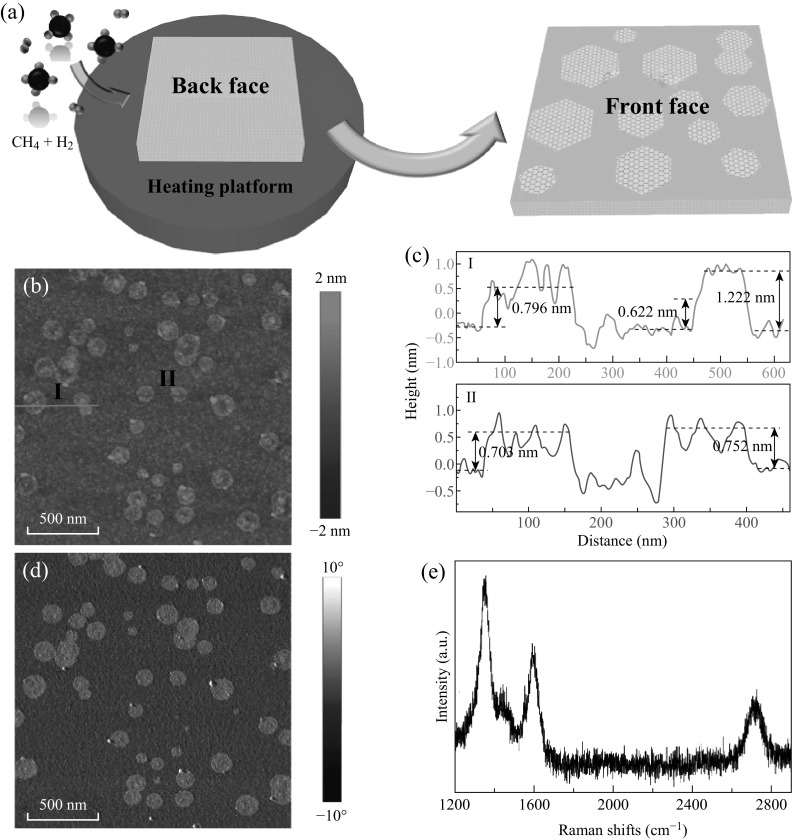
Designed metal-free CVD synthesis and characterization of the as-grown graphene domains on silicon (100) substrates at 910 °C. **a** Schematic diagram of the graphene domains grown on the silicon surface. The substrate was placed upside-down in order to capture more methane molecules during growth, which actually occurs on the front face. **b** Typical AFM height image. **c** Corresponding AFM height profile along the lines I, II in Fig. 1b. **d** Corresponding AFM phase image. **e** Raman spectra of graphene grown on silicon (100) substrates

Figure 1a shows a schematic diagram of the metal-free APCVD equipment used to grow graphene directly on single-crystal silicon. A custom-made, portable chamber with a ceramic wafer was used as the heating platform (Fig. S1). A detailed description of the chamber is also presented in our previous report [35]. The silicon substrate is mounted face down on the heating stage with a narrow gap between the Si and the heating stage. Unlike a commercial CVD chamber for graphene growth, the special design of the chamber used in this work remarkably increases the local concentration of hydrocarbon radicals and C ions, and enhances interaction with the silicon surface when the substrate is placed upside-down. Moreover, the confined hydrocarbon radicals and the increased collision frequency in the narrow gap further facilitate the nucleation of graphene [36]. This assumed mechanism is reasonable because the graphene was only observed on the upside-down surface. On the other hand, conventionally placed substrates cannot produce graphene or carbon nucleation at comparable temperatures.

Figure 1b shows a typical AFM image of a single-crystal silicon (100) surface after graphene growth at 910 °C. After exposure to the reaction gas mixture (CH_4_:H_2_ = 180:10 sccm) for 1 h, the silicon surface was covered with graphene domains. The thickness of the graphene domains was ~ 0.7 nm (Fig. 1c), which is approximately equal to the thickness of a graphene monolayer [27, 28]. Some nearest-neighbor graphene domains can also link together and produce stacked graphene to form a bilayer with a thickness of ~ 1.2 nm (Fig. 1b, c, line I). An XPS full scan of the as-grown sample proves that the growth of graphene on silicon is a complete, metal-free process without any metal contaminants (Fig. S2). Raman spectra were obtained for the single-crystal silicon surface after direct growth. Three prominent peaks were found and assigned at 1353 cm^−1^ (D band), 1592 cm^−1^ (G band), and 2710 cm^−1^ (2D band), confirming the formation of graphene (the prominent D peak should originate from the large number of domain boundaries and H-terminated graphene edges). The weak peak located at ~ 1450 cm^−1^ can be attributed to the C–C stretching and C–H bending vibration [37, 38].

**Fig. 2:**
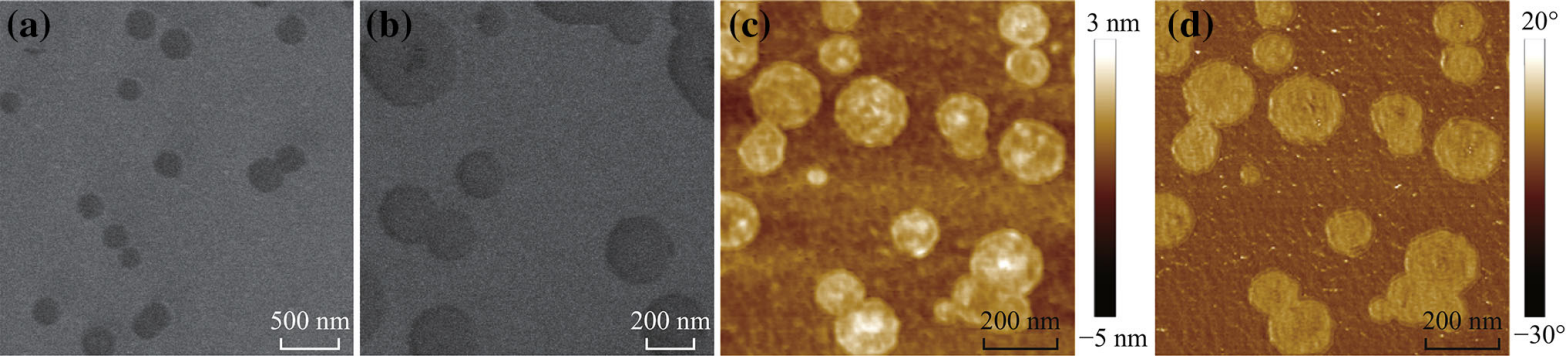
**a**, **b** High-resolution SEM images of the graphene grains on silicon. **c** High-magnification AFM topography and **d** corresponding phase image of the graphene grains on silicon

Figure 2a, b displays the high-resolution SEM images of the graphene grains directly grown on silicon. The graphene grains show a clear contrast difference with the silicon surface, and most of the grains exhibit a regular, hexagon-derived shape, similar to the single-crystal graphene on BN or Si_3_N_4_ substrates [27, 31]. The high-magnification AFM topography and phase image (Fig. 2c, d) also present a similar feature of a hexagon-derived shape, which further indicates the good crystallinity of graphene on silicon.

**Fig. 3:**
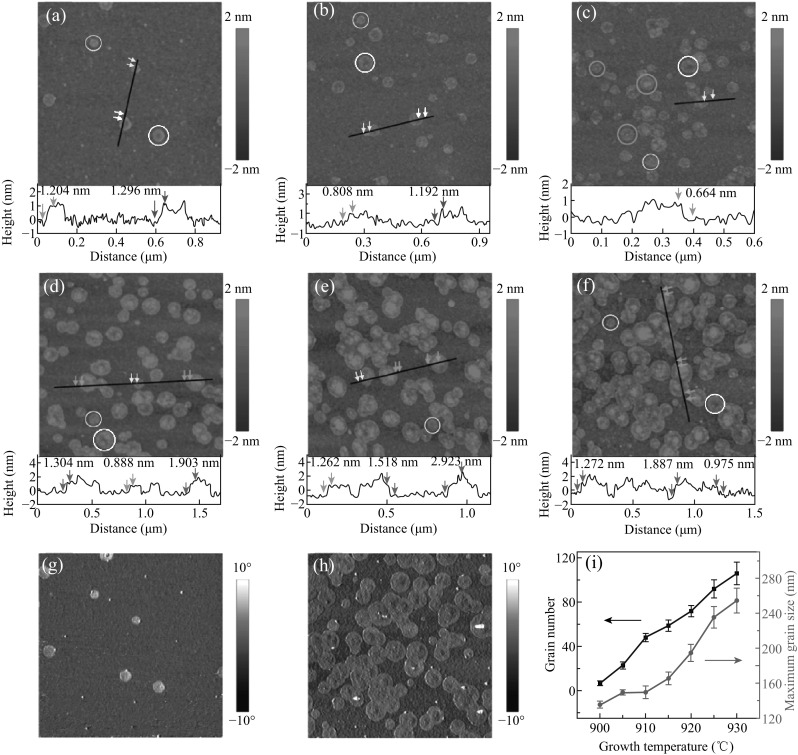
Investigation of graphene nucleation and growth on silicon (100). **a**–**f** AFM height image of graphene domains directly grown on silicon (100) substrates for 1 h at the following temperatures: **a** 900 °C, **b** 905 °C, **c** 915 °C, **d** 920 °C, **e** 925 °C, and **f** 930 °C. The bottom row shows the corresponding height profile along the black line. The arrows indicate the height differences between the corresponding two points. Light blue, blue, and red circles correspond to flat, concave, and bulging graphene domains, respectively. The scan size is 2 µm. **g**, **h** The corresponding AFM phase images of graphene grown at 900 and 930 °C, respectively. **i** The average grain number and the maximum grain size as a function of growth temperature

To better understand the nucleation and growth of graphene on single-crystal silicon, AFM images were recorded for a series of samples grown at temperatures between 900 and 930 °C (Fig. 3a–f). At the relatively low temperatures (900 and 905 °C), only few graphene domains appear (Fig. 3a, b). We can clearly distinguish two kinds of graphene domains, atomically flat mono- or bilayer graphene domains (lateral size of ~ 130 nm) and larger concave bilayer graphene domains (~ 160 nm). These were confirmed by the height profiles shown at the bottom of Fig. 3a, b. At the more elevated temperature (915 °C), the number of graphene domains increases. This is accompanied by larger domain sizes as shown in Fig. 3c. Moreover, only a few core-bulging graphene domains (small convex spots on the center) emerge, which are marked by a red circle. The concave surface still dominates among the graphene grains.

After a further increase in the growth temperature, the core-bulging graphene domains gradually dominate in all AFM images. The height profiles, along the black lines in Fig. 3d–f, clearly show the formation of the core-bulging graphene domains, where the thickness changes from 1.9 to 2.9 nm (3–5 graphene layers). In fact, due to the extremely low roughness (~ 0.3 nm) of the single-crystal silicon surface, three distinct areas are revealed in the AFM height images. These are highlighted by circles and arrows along the height profile lines. The light blue, blue, and red circles and arrows correspond to flat, concave, and bulging graphene domains, respectively. Moreover, even in samples dominated by the bulging domains, small flat monolayer or bilayer domains and concave bilayer domains also exist in the AFM image (light blue and blue circles and arrows in Fig. 3d–f). They show the roadmap of the domain evolution of initially small graphene grains. Figure 3g, h shows the corresponding AFM phase information at 900 and 930 °C. Despite the height variation in the concave bilayer or core-bulging single domain, the AFM phase image reveals a component with a contrast different from that of silicon. This further confirms that the domains are composed of graphene.

The average grain number and the maximum grain size vary as a function of growth temperature in Fig. 3i. Clearly, a higher growth temperature causes a larger domain size and higher nucleation density. This indicates that temperature is a key factor in controlling graphene nucleation and growth. Higher temperatures were also tried for the growth of graphene. However, we found that the surface of silicon was destroyed at temperatures higher than 950 °C (silicon reacts with the active hydrocarbon radicals, Fig. S3). On the other hand, there are other ways of growing larger graphene domains besides increasing the temperature. For example, it has been reported that trace oxygen can effectively accelerate graphene domain growth, for both metallic and dielectric substrates [18, 39]. A further study that explores trace oxygen-aided synthesis of large-area graphene layers is ongoing.

**Fig. 4:**
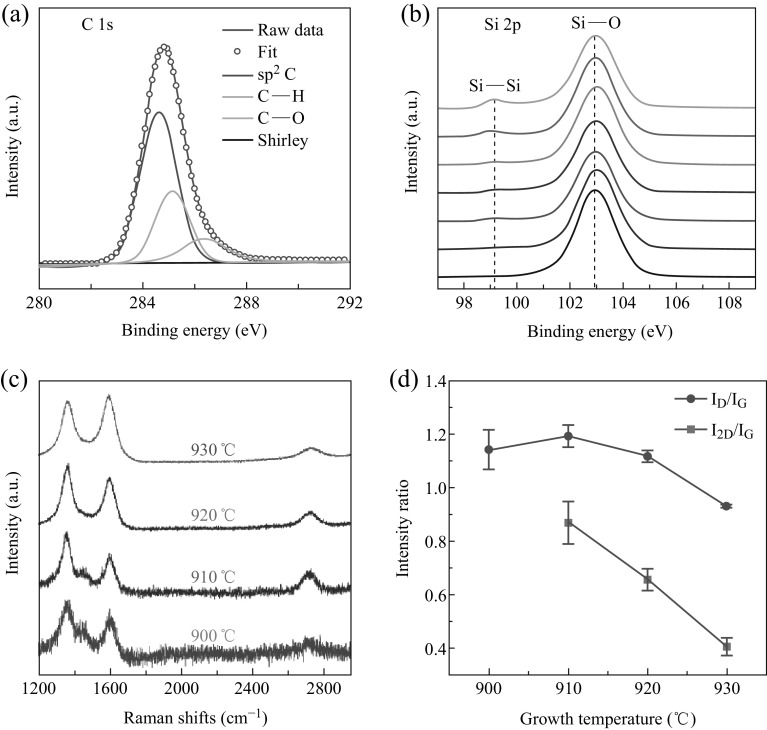
Spectroscopic characteristics of graphene grown directly on silicon. **a** C 1 s XPS spectrum of as-grown graphene. **b** Si 2p XPS spectra of graphene at various growth temperatures, from bottom to top are 900, 905, 910, 915, 920, 925, and 930 °C, respectively. **c** Representative Raman spectra of graphene at various growth temperatures. **d** Ratio of the Raman D band intensity to that of the G band (*I*_D_/*I*_G_), red, and the 2D band intensity to the G band (*I*_2D_/*I*_G_), light blue, as a function of growth temperature

Spectroscopic characterizations were further conducted to probe the microstructural changes in graphene for different growth temperatures. Figure 4a shows the typical C 1 s spectrum for direct growth of graphene on a silicon (100) substrate at 910 °C. This spectrum has been fitted with several peaks assigned to *sp* ^2^ carbon (284.6 eV), C–H (285.1 eV), and a broad C–O (286.4 eV) peak. The dominant peak of the sp^2^ feature confirms the presence of graphene. The C–H (*sp* ^3^ carbon) signal should be due to the H-terminated graphene edges, which is consistent with the Raman spectra in Fig. 1e. In the XPS Si 2p spectra in Fig. 4b, a strong Si–O peak and a weak Si–Si peak appear at ~ 103 and ~ 99 eV, respectively. The Si–O peak is the result of the oxidization of silicon. The oxidization should have occurred only after the silicon substrate was exposed to air because the sample was annealed in a reducing atmosphere. We found that the intensity of the Si–Si peak increases with increasing growth temperature. This should be due to the increasing coverage of the graphene region because graphene prevents the oxidation of Si in air. The lack of the Si–C peak at approximately 100.3 eV indicates that the interaction between the graphene and silicon substrate is weak, and no buffer layer of Si–C was formed during the growth process (Fig. S4). Contrary to the observations of Hackley and Trung [32, 33], the lack of the Si–C peak should be due to the following reason. In our CVD system, carbon is produced by the pyrolysis of methane. Without metallic catalysts, only a small number of C ions with weak mobility and activity were produced. Therefore, they would not react with silicon unless under much higher temperature, as demonstrated by Ki-Bum Kim et al. [34]. The temperature-dependent structure of graphene was further evaluated by Raman spectroscopy, as shown in Fig. 4c, d. Figure 4d shows the changes in the Raman characteristic signals for graphene (the intensity ratio of the D and G band, *I*_D_/*I*_G_, and the intensity ratio of the 2D band to the G band, *I*_2D_/*I*_G_) with varying growth temperatures. The *I*_D_/*I*_G_ ratio is typically used to evaluate the quality of graphene. We found that the *I*_D_/*I*_G_ value increases slightly for graphene growth from 900 to 910 °C. This can be due to the presence of more edges for the dominant concave bilayer graphene domains for samples grown at 910 °C. As the growth temperatures increase to 910 and 930 °C, the enlarged graphene domains (Fig. 3i) lead to a gradual reduction in the *I*_D_/*I*_G_ value. The 2D band to G band ratio (*I*_2D_/*I*_G_) is commonly used as an indicator of the number of graphene layers [40, 41]. The *I*_2D_/*I*_G_ value decreases from 0.87 to 0.41 when the growth temperature increases from 910 to 930 °C (Raman mapping of *I*_2D_/*I*_G_ for the sample grown at 905 °C is shown in Fig. S5). This suggests the layer propagation of graphene domains, which is consistent with our AFM observations.

**Fig. 5:**
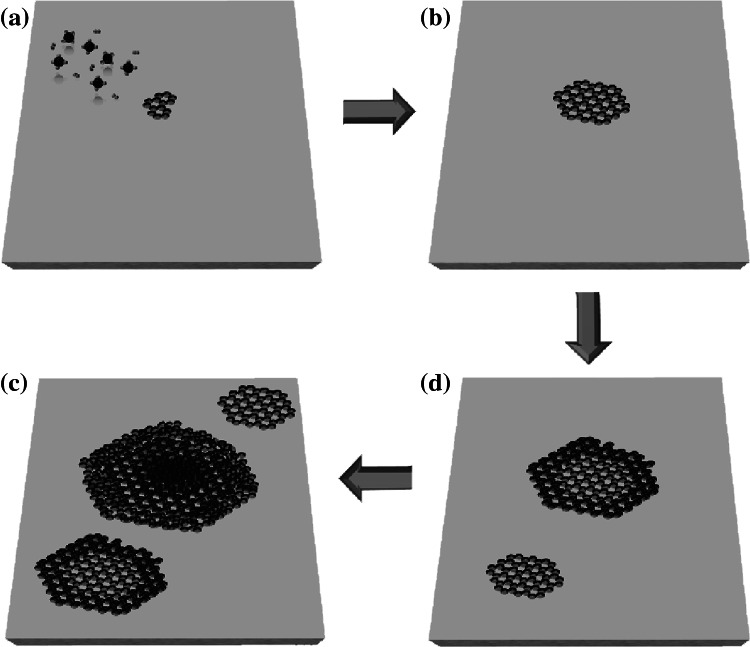
Schematic of the direct nucleation and growth process of graphene domains on silicon substrates using metal-free APCVD. **a** Methane decomposition and super-saturated, active carbon species anchored on the silicon surface. This facilitates graphene nucleation. **b** In-plane propagation with carbon atoms landing at the edge of stable nuclei and the formation of small flat monolayer or bilayer graphene domains. **c** Edge-propagation process for bilayer graphene depositions at the active edge of the monolayer graphene domains, and the formation of larger, concave bilayer graphene domains. **d** Core-propagation process with more graphene layers nucleating in the core of the concave graphene domains, and stabilization of the bigger bulging graphene domains at higher growth temperatures

Based on the above experiments, an integrated growth model considering the nucleation and growth process is proposed. In the first stage, due to the catalytically inert nature of silicon, the thermal self-decomposition of methane produces active carbon species that initiate graphene nucleation (Fig. 5a) [42]. Due to the upside-down-placed substrate, the super-saturation of carbon radicals and high collision frequency promotes the anchoring of carbon atoms onto the silicon surface and facilitates graphene nucleation. It was also reported that the high density of dangling bonds on a silicon surface could enhance nucleation [34, 43]. After the nucleation process, the growth of graphene follows the van der Waals growth model, and the silicon only acts as a support to define the growth plane. In fact, the van der Waals growth model has been successfully applied in growing graphene on insulators [26, 42] and other 2-D materials on silicon [44]. The active carbon decomposed from the continuously supplied methane results in the subsequent growth of graphene (Fig. 5b). The in-plane propagation of graphene initially occurs with carbon atoms landing on the edge of a stable nucleus and forming bonds with the carbon atoms on the edge of the domains. This produces small, flat monolayer, or bilayer, graphene domains. For the monolayer graphene domain, due to the weak interaction between silicon and graphene and the large number of dangling carbon atoms existing at the edge of the domain, the in-plane propagation may be blocked by the formation of C–H bands, which are indicated by the XPS results shown in Fig. 4a [45]. As a result, in-plane propagation stops and the graphene domain reach a critical stable size (~ 130 nm).

At a higher temperature, both the hydrocarbon radicals and the silicon surface should become more active, and an additional layer of graphene forms on the active edge of the monolayer graphene domain. The growth mechanism of graphene changes to edge-propagation instead. Therefore, concave bilayer domains with larger size are formed, as depicted in Fig. 5c. The small bilayer graphene domains, however, do not participate in the edge-propagation process (light blue circle in Fig. 3a, e). This is mainly due to the strong layer interaction in the bilayer and the obtuse nature of the bilayer graphene domain edge [46, 47], which is also confirmed by the stable boundary of the concave bilayer domains. With further increase in growth temperature, the reaction is more active, thus accelerating the propagation process and producing larger graphene domains. Furthermore, superfluous active carbons begin to nucleate in the core of already-grown, concave bilayer graphene domains. The core-propagation process occurs and generates bulging, few-layer graphene domains (Fig. 5d) [30].

The growth of graphene was further explored using different orientations and doping types of single-crystal silicon substrates (including the [100], [111], and [110] surface, and *n*-type and *p*-type silicon). We found that the growth regimes of graphene are independent of the Si surface structure, further indicating that the direct growth of graphene on silicon is a self-propagating process and follows the van der Waals growth model. The growth of graphene on Si is mainly controlled by the temperature and the confined, local environment of the precursors.

## Conclusion

In summary, through the optimization of the growth conditions, we demonstrate the direct growth of graphene onto a single-crystal silicon surface using metal-free APCVD, which is confirmed by AFM, SEM, Raman, and XPS characterizations. In addition, in-plane propagation, edge-propagation, and core-propagation processes were proposed to evaluate the sequentially changing graphene domains. A better understanding of the direct nucleation and growth mechanisms may enable the synthesis of large-area and layer-controlled, high-quality graphene on single-crystal silicon substrates.

## Electronic supplementary material

Below is the link to the electronic supplementary material.

Supplementary material 1 (PDF 635 kb)
