# Supplementary material for: Direct Growth of Graphene on Silicon by Metal-Free Chemical Vapor Deposition
Source: Nanomicro Lett. 2017 Dec 8;10(2):20. doi: 10.1007/s40820-017-0173-1 (PMC6199066; doi:10.1007/s40820-017-0173-1)
Supplement: Supplementary file 1 — Supplementary material 1 (PDF 635 kb) [file 40820_2017_173_MOESM1_ESM.pdf]

Supporting Information for

Direct Growth of Graphene on Silicon by Metal-Free Chemical Vapor Deposition

Lixuan Tai[1, 3, #], Daming Zhu[1, #, *], Xing Liu[1, 2, #], Tieying Yang[1], Lei Wang[1], Rui Wang[1], Sheng Jiang[1], Zhenhua Chen[1], Zhongmin Xu[1], Xiaolong Li[1, *]

[1]Shanghai Synchrotron Radiation Facility, Shanghai Institute of Applied Physics, Chinese Academy of Sciences, Shanghai 201204, People's Republic of China

[2]University of Chinese Academy of Sciences, Beijing 100049, People's Republic of China

[3]Department of Electronic Engineering, Tsinghua University, Beijing 100084, People's Republic of China

[#]These authors contributed equally to this work.

*Corresponding authors. E-mail: zhudaming@sinap.ac.cn, lixiaolong@sinap.ac.cn

Figures

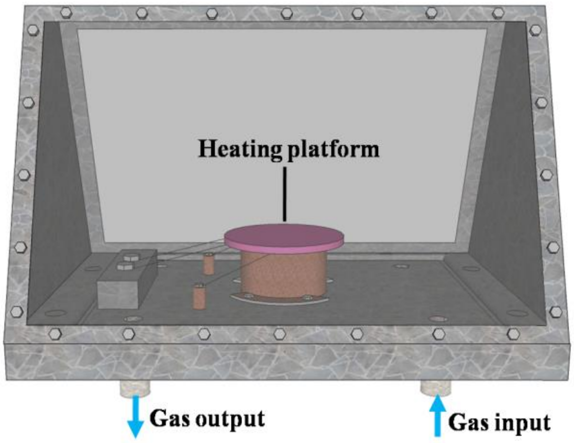

Fig. S1 Sketch of the chemical vapor deposition (CVD) chamber. A well sealed cold-wall CVD chamber with a dedicated built-in heating platform was used for graphene growth

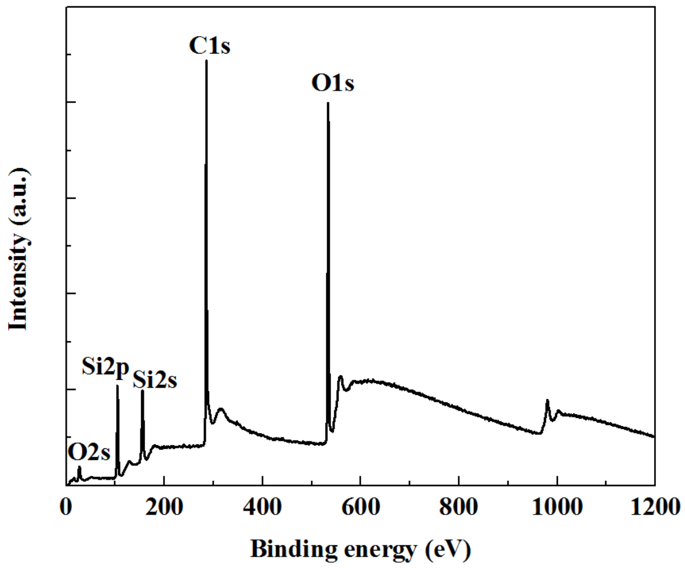

Fig. S2 XPS full scan of the as-grown sample

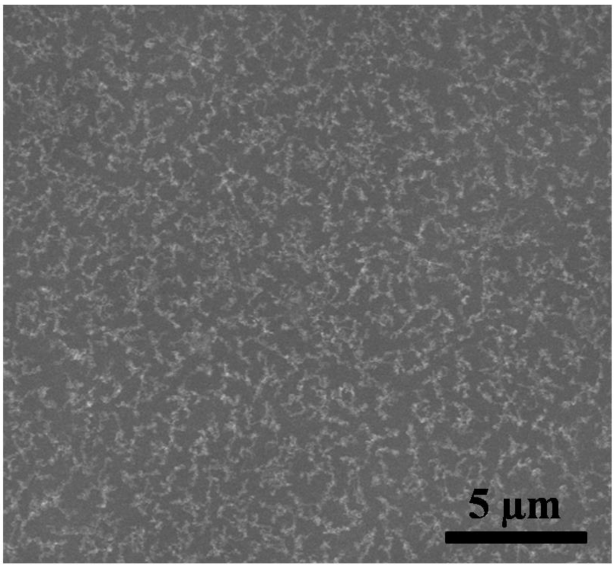

Fig. S3 SEM image of silicon surface after CVD growth at 950 °C. The flat surface of silicon has been destroyed

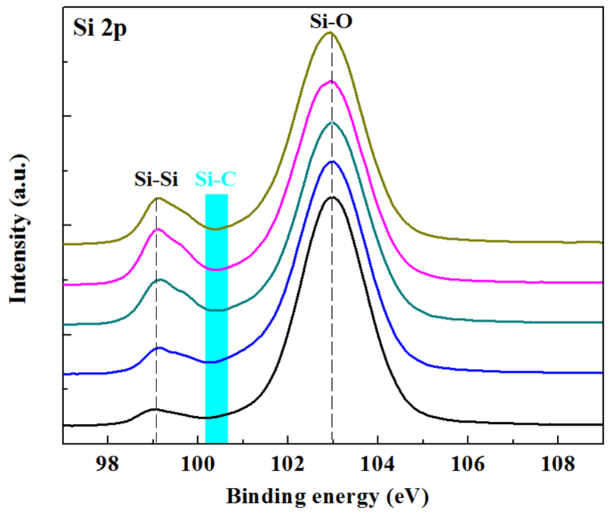

Fig. S4 Si 2p XPS line scan spectra of graphene growth at 935 °C. The interval between every point on the line is 40 μm

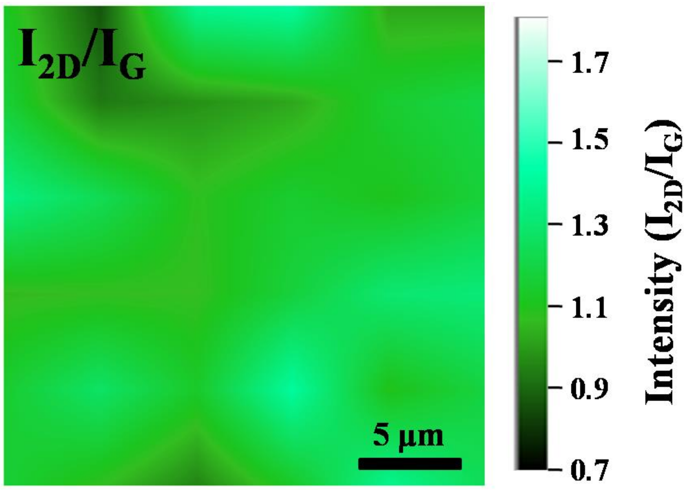

Fig. S5 Raman mapping of the intensity ratio (I_{2D}/I_G) for the sample growth at 905 °C The laser-spot size was about 2 μm with a 473 nm wavelength. The Raman mapping of I_{2D}/I_G over large areas displays uniform distribution (mainly range from 0.9-1.4), implying that the sample is mainly composed of single layer or bilayer graphene domains, consistent with the AFM characterizations.
